# When personal experience becomes professional power: how pre-service teachers discover their creative capacity during teaching internships

**DOI:** 10.3389/fpsyg.2025.1716459

**Published:** 2026-01-13

**Authors:** Al-Khansaa Diab, Edna Green

**Affiliations:** 1 Special Education Department, David Yellin College of Education, Jerusalem, Israel; 2 Students' Dean Office, David Yellin College of Education, Jerusalem, Israel

**Keywords:** pre-service teachers, teaching internships, innovation development, personal-professional integration, teacher education, success narratives

## Abstract

**Introduction:**

Teacher education research has documented beginning teachers' struggles extensively, but we know surprisingly little about the innovative capabilities that emerge during intensive field experiences. This phenomenological study examines how 17 pre-service teachers, 16 women and one man, representing both Arab and Jewish students, aged 22–28, discovered their creative capacity during year-long teaching internships at an Israeli teacher training college. Through interpretative phenomenological analysis of in-depth interviews, we invited participants to share their most meaningful success stories about transformative work with individual students.

**Methods:**

Through interpretative phenomenological analysis, three interconnected themes emerged.

**Results:**

First, participants systematically transformed personal struggles into professional tools for healing, converting difficult life experiences into resources to support students facing similar challenges. Second, they developed sophisticated environmental awareness, paired with methodical relationship-building approaches that evolved from intuitive care into systematic professional practice. Third, they engaged in principled rule-breaking, prioritizing student success over institutional compliance and thoughtfully departing from conventional approaches when these failed to serve student needs. Our analysis reveals that pre-service teachers possess innovative potential that challenges deficit-focused perspectives dominating teacher preparation discourse.

**Discussion:**

This creative capacity flourishes when certain conditions are met: extended time for authentic relationship-building, supervision that encourages personal and professional integration, and institutional environments that support thoughtful innovation. Rather than viewing difficult life experiences as barriers to effective teaching, participants converted personal wisdom into professional resources. These findings suggest that teacher education needs fundamental rethinking. Rather than focusing primarily on fixing deficits, programs could recognize and systematically cultivate the creative power that emerges during intensive field experiences. This requires redesigned supervision approaches, curriculum modifications, and institutional structures that support principled innovation during teaching internships.

## 1 Introduction

Teacher education worldwide grapples with the challenge of preparing effective educators for increasingly complex classrooms. The teaching internship represents the pivotal transformation from theoretical knowledge to practical competence (Flores, 2020), yet institutional challenges persist. While structured internship programmes demonstrate effectiveness in fostering professional growth through authentic classroom experiences (Mantel et al., 2024), systemic inadequacies in mentorship, institutional partnerships, and practical opportunities continue undermining optimal preparation outcomes (Zaman and Mahanta, 2025; Kaur, 2024). These systemic inadequacies reflect a broader challenge facing university-based teacher education programs: how to systematically cultivate innovative capability during extended field placements rather than focusing primarily on deficit remediation.

Recent comprehensive analyses of teacher innovation reveal that pre-service teachers (final-year students completing their teaching practicum before receiving licensure) bring unique perspectives and creative problem-solving capabilities that significantly enhance educational practices when properly supported and recognized (Liu et al., 2024). Research demonstrates that such innovative approaches in higher education contexts enhance efficiency and student motivation (Shalgimbekova et al., 2024). Critical to understanding these innovative capabilities are teachers' personal narratives, which provide insights into practice development and demonstrate the interconnectedness of personal experience and professional growth (Dursun and Aykan, 2025).

The internship period involves intensive professional identity formation as pre-service teachers navigate tensions between pedagogical ideals and classroom realities (Neander Christensson, 2024; Meyer et al., 2023). Recent systematic investigations into teacher identity development have revealed that professional identities emerge through dynamic interactions among personal experiences, institutional contexts, and authentic teaching encounters, rather than through linear skill-acquisition models (Meyer et al., 2023). Research increasingly recognizes teachers' transformative agency during periods of uncertainty, highlighting their capacity for innovation and adaptive responses to challenging circumstances through the creative integration of personal resources with professional knowledge (Chen-Levi et al., 2024).

Professional identity development occurs most significantly during intensive field experiences, where the integration of personal and professional knowledge is explicitly supported by structured reflection and mentoring (Meyer et al., 2023). Contemporary meta-analytical evidence demonstrates that teacher preparation programmes emphasizing innovative practice and global competence achieve stronger outcomes when integrating personal experience with professional development throughout extended field placements, with reported improvements in both teaching effectiveness and professional confidence(Zhang et al., 2024).

These findings support reconceptualizing internships as creative laboratories in which innovative capabilities emerge through dynamic interactions among personal insight, professional knowledge, and authentic student needs.

Despite growing recognition of pre-service teachers' innovative potential, limited research examines what characterizes pre-service teachers' most meaningful success experiences and how these experiences reveal innovative capabilities during intensive internship placements. Recent comprehensive reviews of teacher innovation research reveal that, while the field has expanded significantly, with over 67% of studies published between 2018 and 2023, conceptual clarity remains limited regarding how innovation actually develops during teacher preparation (Liu et al., 2024). Most existing studies focus on either personal characteristics or environmental factors in isolation, without examining their dynamic integration in authentic teaching experiences.

This gap is particularly significant because understanding the nature of transformative success experiences has crucial implications for the design of teacher education programmes. Contemporary research demonstrates that when programmes explicitly support the integration of personal experience with professional practice, pre-service teachers develop enhanced creative problem-solving capabilities, stronger professional identities, and more effective teaching practices (Fitzsimons et al., 2024; Meyer et al., 2023). However, the lived experiences underlying these transformative successes—how pre-service teachers actually experience and make meaning of their most significant teaching achievements—remain underexplored.

Furthermore, existing research often treats innovation as a technical competency rather than examining it as a lived experience involving the whole person. Recent theoretical developments emphasize the importance of understanding how teachers' personal narratives, cultural backgrounds, and life experiences become integrated with professional knowledge during intensive field experiences (Neander Christensson, 2024). This integration process appears particularly crucial for developing the kind of responsive, culturally sensitive, and innovative teaching practices needed in today's diverse classrooms.

This qualitative phenomenological study addresses these knowledge gaps by exploring the most meaningful success experiences shared by pre-service teachers during college internship placements. The phenomenological approach enables a deep examination of the lived experiences underlying successful transformative teaching, providing insights that quantitative measures of teaching competence cannot capture. Understanding how pre-service teachers make meaning of their greatest achievements has significant implications for teacher education programmes seeking to cultivate innovative practitioners rather than merely competent technicians.

The study contributes to teacher preparation literature by examining what characterizes transformative success experiences and revealing the innovative capabilities that emerge within these narratives. By analyzing how pre-service teachers describe their most meaningful internship successes, this research provides frameworks for supporting the development of transformative practice. The findings offer practical implications for programme design, supervision practices, and mentoring approaches that can better support the innovative potential evident in successful internship experiences. The contemporary relevance of this research is underscored by the current educational challenges that require innovative teaching approaches. Recent global disruptions have underscored the need for teachers who can adapt creatively to changing circumstances while maintaining a focus on student success (Chen-Levi et al., 2024). Teacher education programmes worldwide are seeking evidence-based approaches for developing such capabilities, making this investigation of success experiences during internship placements particularly timely and significant. Academic developers and field supervisors can immediately redesign supervision approaches to recognize and assess principled innovation, rather than merely compliance with predetermined practices.

### 1.1 Conceptualizing innovation in this study

Before we proceed, we should clarify what we mean by “innovation” in this context, since the term carries different meanings across educational literature. We are examining how pre-service teachers develop the capacity to respond creatively when conventional approaches fail their students. We distinguish between three related concepts: innovative capability (pre-service teachers' potential for creative problem-solving, innovative teaching approaches (the specific classroom practices documented in their success stories), and innovation development (the process by which this creative capacity emerges during internships). This shifts attention from innovation as product to innovation as process; we are interested in how pre-service teachers transform from rule-following beginners into practitioners who can respond thoughtfully to complex student needs.

This study examines what happens when pre-service teachers succeed during them internships rather than when they struggle. By analyzing participants' most meaningful success stories, breakthrough moments with individual students, we aimed to understand how personal experiences transform into professional capabilities, and what contextual factors enable creative problem-solving during intensive field experiences.

Research questions:

1. How do innovative teaching approaches manifest in the success stories shared by pre-service teachers during teaching internships?
2. What personal and contextual factors enable the transformative practices described in pre-service teachers' success narratives?

## 2 Theoretical framework

This study employs three complementary theoretical frameworks to examine how pre-service teachers develop innovative teaching approaches during college internships. This integrated approach recognizes that creative practice emerges through complex interactions between contextual conditions (Ecological Systems Theory), psychological empowerment (Self-Determination Theory), and passionate commitment (Educational Eros), rather than examining these factors in isolation.

### 2.1 Ecological systems theory

Bronfenbrenner's (1979) ecological framework provides a comprehensive lens for understanding how environmental layers shape the emergence of innovative practices during internships. Contemporary research confirms that this bioecological perspective remains “an ideal framework for understanding how individuals negotiate the dynamic environment and their own identities in international and intercultural education settings” (Tong and An, 2024). However, recent systematic reviews indicate that most studies either adopt the earlier version of the theory or present an incomplete bioecological framework, which “oversimplifies the richness of Bronfenbrenner's theory and does not sufficiently demonstrate its value” for understanding complex educational processes (Tong and An, 2024). Ongoing investigations validate the ecological theory's relevance across diverse educational settings (Amali et al., 2023). Recent applications demonstrate the theory's continued relevance across diverse educational contexts. Research on school belonging utilizes the bioecological framework to explain how “the development of a feeling of belonging at school occurs because of different interactions between developing students and the school's ecological environment over time” (El Zaatari and Maalouf, 2022). This approach emphasizes the Process, Person, Context, and Time (PPCT) model, in which proximal processes are the primary mechanisms of development.

In teacher education contexts, the microsystem encompasses immediate relationships between pre-service teachers, students, cooperating teachers, and college supervisors that directly influence willingness to experiment with innovative approaches. Recent research confirms that “every system mentioned in the Ecological Systems Theory plays an important role in students' motivation in learning” (Perera, 2023). The mesosystem represents college-school partnerships that create supportive conditions for innovation, while the exosystem includes broader institutional policies and community resources that indirectly shape internship experiences.

Contemporary scholars have called for more sophisticated applications that examine system interactions rather than isolated components. Higher education research demonstrates how ecological models can “map knowledge” and “identify recommendations at the micro- and meso-systemic levels” (Renn and Smith, 2023). The theory's evolution toward bioecological perspectives emphasizes that ongoing, complex interactions between individuals and their environments (proximal processes) are the primary drivers of development, with recent research confirming that “various ecological system levels influence children's cognitive, emotional, and social development, and explores the impact of these systems' interactions on overall child growth” (Tong and An, 2024).

### 2.2 Self-determination theory

Deci and Ryan's Self-Determination Theory (SDT) explains how the universal needs for autonomy, competence, and relatedness foster motivation and growth (Deci and Ryan, 2000; Ryan and Deci, 2020). Recent research in teacher education has shown that these needs evolve during practicum experiences (Gidalevich and Shalev, 2022), shape pre-service teachers' intentions to use autonomy-supportive practices (Tan and Levesque-Bristol, 2024), and can be strengthened through SDT-based interventions that enhance readiness for classroom teaching (Großmann et al., 2023). Together, these studies highlight SDT's relevance for supporting innovation and identity development in teacher preparation programs.

Recent meta-analyses demonstrate robust empirical support for SDT applications in educational contexts, showing that autonomous motivation leads to positive educational outcomes and that satisfaction of competence, autonomy, and relatedness needs promotes autonomous motivation (Guay, 2022). These findings are particularly relevant to understanding how supportive internship environments can nurture creative teaching approaches during teacher preparation.

Contemporary applications in teacher education reveal sophisticated relationships between need satisfaction and professional development. The three basic needs work synergistically to enhance motivation (Howard et al., 2020).

Research demonstrates that fulfillment of SDT needs enhances pre-service teachers' adaptability, task engagement, and professional confidence (Gidalevich and Shalev, 2022). Teacher candidates particularly value relationships with peers, mentor teachers, and university advisors as sources of relatedness support as they develop competence and autonomy (Tan and Levesque-Bristol, 2024).

Autonomy has gained attention in teacher preparation, as feeling autonomous enables teachers to implement practices aligned with their values. SDT-based interventions can strengthen these psychological needs, improve pre-service teachers' readiness, and support their intentions to use autonomy-supportive practices with students (Großmann et al., 2023). When teachers feel competent, they adopt more student-centered approaches and provide greater choice and scaffolding.

### 2.3 Educational eros

Educational Eros provides the motivational foundation that transforms technical competence into transformative practice within both ecological contexts and psychological empowerment frameworks. Unlike mere professional caring, Educational Eros embodies a passionate commitment to student growth, educational excellence, and authentic engagement with the vulnerable dimensions of teaching (Tsabar, 2014; Hattie, 2003).

Recent systematic reviews reveal that passionate teaching involves complex combinations of different love dimensions, with contemporary research identifying “four distinct notions of passion: i.e., romantic, friendly, erotic, and divine love” that range “from love of the Truth and the Good to a calling from the subject” (Jons, 2024). These findings emphasize that Educational Eros is not merely an emotional state but a systematic approach to professional practice.

Contemporary research emphasizes the developmental nature of teacher passion. Recent studies demonstrate that passionate teachers invest extraordinary time and energy into instruction, capturing students' attention, and that teachers can cultivate greater harmonious passion through autonomy-supportive workshops and the incorporation of intrinsic instructional goals (Jang et al., 2023). Furthermore, teachers expressed emotions to transmit their passion to students, fostering engagement and motivation (Chichekian et al., 2024). These findings highlight the importance of both teacher development and classroom practices in creating passionate learning environments.

The relationship between teacher passion and student outcomes is supported by substantial empirical evidence. Passionate teachers serve as mentors and role models whose enthusiasm fosters student engagement and achievement, and harmonious passion is linked to greater teacher satisfaction, lower burnout, and improved student classroom behavior (Carbonneau et al., 2008). Recent evidence suggests that students' passion for learning is significantly influenced by their perceptions of teacher passion, particularly when combined with supportive learning environments (Chichekian et al., 2025). Passionate teachers cultivate sustained student motivation and interest in learning (Serin, 2024), underscoring the transformative influence of teacher passion on academic and personal growth.

Educational Eros manifests through specific professional behaviors—systematic relationship-building, authentic engagement with students' needs, and a willingness to be professionally vulnerable in the service of student growth—rather than merely as personal qualities. Research confirms that professional vulnerability is central to authentic development and relational engagement, reshaping teacher identity and deepening commitment to student learning (Holappa et al., 2021; Bacova and Turner, 2023).

The integration of these three frameworks addresses a gap in research that typically employs single theoretical lenses, which may not capture the multidimensional nature of professional development. Recent research supports this integrated approach, demonstrating that emotional management is integral to teachers' professional work within specific cultural contexts (Lai et al., 2024). This integration suggests that transformative teaching practices emerge through environmental support, psychological empowerment, and passionate commitment, working synergistically during authentic internship experiences.

Integration of these frameworks reveals how innovation develops during teacher preparation. Supportive environments, characterized by extended time for relationship-building, mentoring relationships that encourage experimentation, and institutional tolerance for appropriate risk-taking, create conditions in which pre-service teachers feel safe to innovate. Within such contexts, the psychological needs emphasized by Self-Determination Theory become relevant: autonomy enables the principled rule-breaking evident in our narratives, competence develops through successful student breakthroughs, and relatedness deepens through intensive one-on-one work. Educational Eros supplies the crucial motivational element, the passionate commitment to individual student success that transforms possibility into action. Our findings suggest that innovation emerges not from environmental support alone, nor from psychological readiness alone, but from their interaction with deep care for students.

### 2.4 Integration of frameworks

These three frameworks work together rather than separately. Ecological Systems Theory directed our attention to environmental conditions in the data, temporal structures, supervisory relationships, and institutional supports that enabled or constrained innovation. Self-Determination Theory helped us identify when participants' narratives revealed autonomy (departing from conventional approaches), competence (confidence evidenced by student breakthroughs), and relatedness (deep connections through intensive relationships). Educational Eros provided language for the passionate commitment we saw driving vulnerable, persistent work with students. During analysis, we asked three questions of each narrative: What environmental factors enabled this innovation? What psychological needs were being satisfied? What evidence of passionate commitment drove these actions? This multi-lens approach precluded single-factor explanations and revealed that innovation emerges from the dynamic interaction among supportive contexts, psychological empowerment, and passionate commitment to student well-being.

## 3 Methods

This study employed a phenomenological approach to explore how pre-service teachers discover and express their creative power during college teaching internships. Phenomenological methodology focuses on understanding lived experiences and the meanings individuals assign to these experiences (Alase, 2017), making it an appropriate approach for investigating the transformative internship period during which student teachers develop innovative teaching approaches through authentic classroom engagement.

### 3.1 Research design

This study presents a focused analysis of participants whose success narratives centered on transformative relationships with challenging individual students, drawn from our larger investigation of pre-service teacher support systems (Diab and Green, 2024). This focused selection enables phenomenological examination of innovation development within intensive one-on-one relationships, revealing patterns distinct from narratives about general classroom management or systemic achievements. The theoretical frameworks of Ecological Systems Theory (Bronfenbrenner, 1979), Self-Determination Theory (Deci and Ryan, 2000), and Educational Eros (Tsabar, 2014) provided analytical lenses for understanding the development of innovation as articulated in participants' narratives.

### 3.2 Context and participants

This study took place at an Israeli teacher-training college serving diverse Arab and Jewish student populations in preparation for elementary education. The final-year teaching internship entails a comprehensive, year-long field experience with gradually increasing teaching responsibilities, supported by ongoing mentorship.

Our original study began with an open call to fourth-year students, inviting them to share success stories from their internship experiences. Fifty-one students responded and participated in comprehensive interviews. In this dataset, the current analysis focuses on 17 participants whose narratives centered on breakthrough moments with individual challenging students rather than on systemic achievements or general classroom success.

We selected these 17 participants through narrative content analysis, in which the central story centered on transformative work with a particular student facing significant challenges. Selection criteria included: evidence of transforming personal experience into professional capability, development from conventional to innovative approaches, detailed relationship-building accounts with individual students, and creative pedagogical solutions emerging through authentic classroom challenges.

The final participant group consisted of 16 women and one man, aged 22 to 28 years, representing diverse cultural backgrounds, including both Arab and Jewish students from various socioeconomic backgrounds. Our selection of 17 participants reflects principles of phenomenological sampling, in which depth is prioritized over numerical saturation. By our 15th interview, we noticed remarkable coherence in transformation patterns; the final two participants confirmed rather than contradicted existing themes, adding nuance rather than entirely new insights. We focused on these 17 stories because they provided rich, detailed accounts of breakthrough moments with individual students, giving us the depth needed to understand how innovation develops.

All completed year-long teaching internships in diverse settings. All participant names used in the following narratives are pseudonyms to protect confidentiality.

### 3.3 Data collection

Data collection for the larger study (Diab and Green, 2024) involved semi-structured interviews conducted at the end of the internship year when participants had sufficient experience for meaningful reflection. Interviews lasted 60–90 min, allowing for a thorough exploration of their most meaningful internship experiences and the factors that contributed to their success.

Interviews were conducted in participants' native languages (Hebrew for Jewish students, Arabic for Arab students) to ensure authentic cultural expression. This approach enabled participants to articulate lived experiences comfortably in their mother tongue, capturing cultural nuances and emotional expressions.

The interview protocol invited participants to share their most significant success stories, exploring the challenges they faced, the approaches they developed, and the relationships they built with students. Probing questions encouraged detailed elaboration of transformative moments, innovative solutions, and integration of personal experiences with professional practice. Interviews in Arabic or Hebrew were translated into English with Claude AI and checked by the first author (fluent in both languages) to ensure accuracy and cultural nuance.

### 3.4 Data analysis

Data analysis followed Braun and Clarke's (2019) reflexive thematic analysis approach, using their six-phase process to explore how innovation and creativity develop during internship experiences.

We began with familiarization (reading all transcripts multiple times, noting significant moments). The coding phase involved identifying meaning units related to innovation and personal-professional integration. We then generated initial themes by grouping related codes and identifying broader patterns. During the reviewing phase, we checked themes against transcripts, refining boundaries and dropping themes that did not hold up. Defining and naming themes required articulating each theme's essence;

For example, “Personal Experience as Professional Resource” captures systematic transformation, not just difficult experiences. Finally, we selected exemplar quotes that illustrated themes while weaving in theoretical analysis. Throughout these phases, our three theoretical frameworks provided interpretive lenses. When we saw environmental factors, the ecological theory helped us understand their significance. Self-Determination Theory provided a framework for autonomy, competence, and relatedness. Educational Eros helped us recognize passionate commitment as a systematic practice rather than a personality.

We used constant comparative analysis, moving iteratively between individual narratives and emerging patterns to ensure themes captured both unique personal experiences and systematic elements characterizing the development of innovative practice (Robinson and Williams, 2024). This systematic analytical approach enabled the identification of key themes illuminating pre-service teachers' lived experiences during their creative transformation journey. Each emergent theme was rigorously examined for phenomenological grounding through detailed analysis of participant narratives and supporting exemplar quotations that captured the essence of their innovative practice development.

### 3.5 Researcher positionality

The first author's background as a Palestinian Muslim educator and the second author's perspective as a Jewish Israeli educator facilitated a culturally sensitive analysis of the experiences of Arab and Jewish students. The first author's fluency in both Arabic and Hebrew proved crucial for accurate translation. Regular discussions between co-authors facilitated reflexive examination of interpretive choices throughout the analytical process.

### 3.6 Trustworthiness and ethics

Credibility was established through prolonged engagement with data, systematic analytical procedures, and ongoing discussions between co-authors with complementary cultural perspectives (Lim, 2025). Dependability was maintained through detailed analytical memos that documented the evolution of themes and interpretive choices. Confirmability was supported through transparent reporting of theme emergence from participant narratives, accompanied by exemplar quotations. Transferability was enhanced by rich contextual descriptions, enabling readers to evaluate its relevance to their own contexts.

This focused analysis utilized the same ethical approval and participant consent as the original study, as both papers examine the same core phenomenon of internship success experiences. Confidentiality was maintained by using pseudonyms and removing identifying information. All participants understood their status as volunteers and their right to withdraw without academic consequences.

## 4 Findings

The analysis of pre-service teachers' success experiences during their college teaching internship revealed three interconnected patterns that emerged consistently across participants' narratives. These patterns illuminate the systematic processes through which student teachers discover and express their creative power during authentic classroom engagement, revealing transformations that challenge deficit-focused perspectives on beginning teacher development. All participant names used in the following narratives are pseudonyms to protect confidentiality.

### 4.1 Personal experience as a professional resource

Across participant narratives, a striking pattern emerged: personal struggles and traumatic experiences systematically transformed into professional strengths and innovative teaching approaches. This transformation process reveals both how innovation manifests in practice and the personal factors that enable creative problem-solving during intensive field experiences.

#### 4.1.1 Vulnerability as professional strength

Multiple participants discovered that their most difficult life experiences became their most powerful professional tools. Hila's experience illustrates this pattern clearly. Having lost her father in childhood during military service, she initially considered this personal history irrelevant to her teaching practice. When confronted with a student who had suddenly lost his father, she made a pivotal decision:

“The father of Avi, one of the kids, died of a heart attack at age 32... One morning, he just felt unwell... before his son woke up... they took him to the hospital, and he passed away on the way. The boy came to me in the morning as usual, and then they notified me that his father had died... Dealing with having to go into the kindergarten smiling like everything is fine and normal, and make some switch... You know... when I was a child, I lost my father in the army services, I was around the same age... So I also told the boy that I had lost my dad. I asked him to tell me about things he liked to do with his dad, and also what I loved, to show him that we actually went through the same thing and that I am okay now, and I am grown up, and I smile and laugh and play, meaning everything continues even though we miss Dad... Moreover, we had a good connection.”

This moment of vulnerability could have gone poorly, as sharing personal trauma with a grieving child crosses boundaries that teacher education typically warns against. However, note what Hila actually did: she did not merely share her pain. She shared her survival, her continued capacity to “smile and laugh and play.” She was offering a map through grief, drawn from her own journey. This distinction matters because it reveals vulnerability as a strategic professional practice rather than emotional oversharing.

What makes this truly innovative rather than simply compassionate is how Hila transformed this individual connection into a systematic pedagogical practice:

“We wrote a letter to Dad every day in kindergarten. It was not easy; it was painful. The child longed for his father and worried about his mother, who always cried. At the end of the year, we compiled all the letters and everything he had told us about his dad and brought it to his mom and him. It was difficult for all the kindergarten children. They buried many toys, and in the doll's corner, they talked about ‘Mom died' and ‘Dad died.' They even told their parents that my dad had died, too. However, we move on. Having time every day for him to talk about his dad helped him get through the day as usual, play, be happy, and make friends with the kids. That is important. I projected this strongly, and Avi grasped that. I say he was lucky I was in the kindergarten because I understood him, and from a similar place, I could help him.”

Hila's approach involved a daily ritual (not occasional or random support), structured emotional processing through literacy activities, documentation of the healing journey, family involvement through the compilation of letters, and community-wide opportunities for processing loss. This was not intuitive caring; it was methodical, professional practice grounded in personal understanding. Her explicit recognition that “he was lucky I was in kindergarten because I understood him” reveals awareness that personal experience had become a systematic professional resource.

#### 4.1.2 Childhood adversity as teaching insight

This pattern of transforming personal wounds into professional tools for healing was not unique to Hila. We observed it repeatedly, though it was expressed through different experiences and contexts. Lili's experience demonstrates how childhood anxiety and exclusion became resources for supporting similar students:

“I had a boy who could not sit still during group work...he would wander off behind the cabinets, play alone...From his mother's stories, I understood that they had given up on him in his previous preschool. It was more convenient for the teacher for him to play quietly behind the cubbies. He was a very fearful child. Everyone else built blocks, and he built guns. His drawings often featured guns, or everything was painted in a very dark blue or black, regardless of what was drawn on the page. My staff and I discovered he was in a constant state of fear and anxiety. He told us he was underground, and snakes and crocodiles were waiting to bite him and his parents.”

Lili recognized this child's isolation and fear, in part, because she had experienced them herself. Her explicit connection between her own childhood experiences and her professional capability reveals the conscious nature of this transformation:

“...as a child, I lacked confidence about many things – whether participating in class or not. When I participated, the teacher would tell me it was non-sense. Most teachers put me down and did not like me. I also did not have good friends. I think what I went through as a child helped me understand these kids and help them...”

However, understanding alone does not constitute innovation. What makes Lili's approach remarkable is how she translated personal insight into a systematic intervention that mobilized the entire classroom community:

“There was teamwork here to free him of his fears; even the children helped him. Initially, they participated in his gun-and-soldier play and later mediated by suggesting a shift to other games, such as animal and house games. Then, a friend he already trusted invited him to play with him. It was wonderful to see that.”

Lili orchestrated peer involvement, gradual transition from fear-based to healthier play, and strategic relationship-building, all grounded in her recognition of what she had needed as an anxious, excluded child. This demonstrates how personal experience becomes professional methodology when combined with thoughtful intervention design.

#### 4.1.3 Confronting personal bias as innovation catalyst

Sometimes the personal experience that becomes a professional resource is not past trauma but present bias that requires confrontation. Flora's journey reveals this less comfortable dimension of personal-professional transformation. Working in a Jewish-Arab kindergarten, she initially experienced significant discomfort:

“I had one child I was very, very wary of; at first, he was an Arab child.”

Later, she elaborates: “It was not due to a language barrier since I succeeded in communicating via pantomime, but rather his violent behavior and use of ‘terrorist suicide bomber' costumes.”

Flora's honesty about her initial wariness is remarkably different from what most pre-service teachers might hide. However, this vulnerability enabled genuine transformation. Through sustained engagement and presumably supportive supervision, her fear shifted:

“I cope with him as back-and-forth attempts of approaching and distancing from him, which ultimately helped me see his distress as ‘a child like all children with many struggles desperately needing warmth and love.' With this insight, I successfully reached out and met his needs. She describes in detail the process of connecting with him and developing his abilities. Flora adds about her relationship with the child's father and how she helped him accept the child and see what makes him special.”

Flora's “back-and-forth attempts” reveal the messy reality of confronting bias; it was not a single epiphany but gradual work. Her innovation involved cross-cultural bridge-building with both child and family, moving from fear to genuine advocacy. The transformation of personal bias into professional cultural competence demonstrates how even uncomfortable personal experiences can become professional resources when supported appropriately.

#### 4.1.4 Explicit recognition of shared experience

Working with adults who have learning difficulties and severe behavioral problems, Jameel discovered that daily storytelling became his most effective teaching tool. He read real-life stories that inspired motivation to learn and succeed, and observed that this simple strategy helped his students overcome challenging behaviors and academic struggles that had persisted for years. What made Jameel's approach particularly compelling was his explicit articulation of how personal transformation becomes professional capability. His remarkable self-awareness demonstrates the universality of this pattern across different contexts and populations, extending beyond childhood education to adult learning environments:

“This success did not come from out of the blue...I did not expect it to be so hard...I succeeded because I see myself in those pupils - I was like them, a troublemaker kid who spent most of his time outside the class. After undergoing a profound transformation, I am now more flexible, tolerant, and realistic, and I can better navigate life's realities. I have strength and hope. Thank God my investment in those kids was not wasted.”

Jameel's explicit articulation, “I see myself in those pupils,” captures what the other participants demonstrated implicitly. He names the process: personal transformation becomes professional capability not automatically, but through conscious recognition and deliberate application. His story also extends the pattern beyond early childhood contexts, suggesting that personal-professional integration operates across different educational levels and student populations.

Across these four narratives, a consistent transformation process emerges. Pre-service teachers recognized parallels between their own struggles and their students' challenges, deliberately deployed personal understanding as a professional resource, developed systematic approaches grounded in that understanding, and explicitly recognized (at least retrospectively) how personal experience had become professional strength. This pattern fundamentally challenges the assumption that difficult life experiences constitute barriers to effective teaching. Instead, participants discovered that their capacity to reach struggling students emerged not despite their personal struggles, but because of them—when supportive environments encouraged such personal-professional integration.

### 4.2 Contextual intelligence and relational pedagogy

A second theme emerged across participants: the development of sophisticated contextual awareness, coupled with systematic approaches to relationship building and problem solving. This theme reveals how innovation manifests through the integration of environmental sensitivity with methodical relational practices.

#### 4.2.1 Reading complex social and emotional contexts

Multiple participants developed exceptional abilities to read subtle contextual cues and respond with advanced interventions. Hiba's experience with a 4-year-old with sensory processing difficulties demonstrates this pattern. Working with a child whose mother was seriously ill, she recognized the complex layers affecting his behavior:

“…His father brings him to kindergarten and picks him up. Despite his difficulties, I had an immediate connection with him because I was also such a child - my mother was sick, and my father took care of us. I knew what I needed for myself, and that is how I acted with him. I explained to the children that it is tough for Sami in kindergarten. I called his disability by name and explained why he falls or drops objects. I also talked about Sami's mother and how we should pray for her recovery, but right now it is hard for Sami because he is worried about his mom... As a result of this open dialogue, the children shared with me aspects they are not good at and concerns about events at home. They really opened their hearts to me…”

Hiba's approach reveals a crucial aspect of contextual intelligence; it is not merely about understanding individual factors but about recognizing how multiple systems intersect. She saw that Sami's difficulties were not solely about sensory processing or family illness or peer relationships, but about how all three layered together. Her response was equally sophisticated: rather than addressing each issue separately, she created a transparent community dialogue that addressed all dimensions simultaneously. By naming Sami's disability explicitly and connecting it to his mother's illness, she gave classmates a framework for understanding rather than judging his behavior.

The transformation this produced demonstrates the power of contextual intelligence paired with deliberate relational practice. Sami's classmates began actively including him, offering assistance during sensory struggles, and showing patience during challenging moments. The child who had been isolated became engaged, developed genuine friendships, and gained confidence as peer understanding replaced judgment. This was not accidental; it resulted from Hiba's systematic reading of complex contexts and her methodical creation of an inclusive community.

#### 4.2.2 Cultural and linguistic responsiveness

Contextual intelligence also operates across cultural and linguistic dimensions. Salam's experience working with Irena, a 5-year-old Russian girl with language difficulties and aggressive behavior, illustrates how innovation emerges when pre-service teachers recognize needs that others miss:

“.. as a mix of Palestinian father and Russian mom, I recall my grandmother's Russian songs calming me as a child… I recorded Irena singing in Russian and played it for her during difficult moments. The song deeply touched Irena, who hugged the phone tightly and shed a tear alongside me. I was silently amazed by the profound solution.”

Salam's innovation demonstrates contextual intelligence operating at cultural and emotional levels simultaneously. While others identified language difficulties and aggressive behavior requiring management, Salam recognized cultural and linguistic disconnection as a source of distress. Her response, which included a recording of Irena singing in Russian, was not merely culturally sensitive. It was strategically designed to address the root issue: a child's sense of disconnection from her cultural identity in a predominantly Arabic-speaking environment.

The phrase “I was silently amazed by the profound solution” captures an important point. Salam herself seemed surprised by how powerfully this simple intervention worked, suggesting that contextual intelligence sometimes operates intuitively before becoming conscious methodology. Her approach integrated family culture with professional practice, transforming cultural sensitivity from an abstract principle into a concrete, systematic intervention.

#### 4.2.3 Systematic relational practice

Rania's experience with at-risk adolescent girls reveals how contextual intelligence operates in particularly challenging social contexts. When she encountered immediate hostility on her first day, her response demonstrated sophisticated environmental reading:

“When I first came to teach, the student Reem approached me and said:...We do not want you here... I do not want to come to your class. I knew she was a good student and that something had happened to her this past year. So, I said, ‘No problem; I would not force anyone to attend my class.' I do not take attendance... Those who want to come and enjoy coming are welcome. Otherwise, it is also fine.”

Rania's contextual intelligence enabled her to read rejection not as personal hostility but as evidence of previous negative experiences. Her response, explicitly giving students autonomy rather than asserting authority, addressed what she had diagnosed: these students had been controlled, judged, and coerced by previous teachers. By removing coercion entirely, she created the psychological safety necessary for authentic engagement.

The immediate results validate her reading of the context:

“I started with an introduction and a short conversation. When class ended, and the students went on break, Reem took me aside and said: ‘I have had terrible days because of the other teacher (we did not get along)... I am sorry I told you I do not want to learn… I promise I will never skip your class based on how you treat me.'... Moreover, she did not skip a single lesson, and significant positive behavioral changes occurred.”

However, contextual intelligence alone does not explain Reem's transformation. Rania paired environmental reading with systematic relational pedagogy that became increasingly sophisticated over time:

“After 2 months, the principal came multiple times to ask me what exactly I did with this student. The girl began to care about her studies again, achieving high grades... I received feedback from her teachers that she has begun reading more, has greater self-confidence, and has started participating in class, even when she risks being wrong. I succeeded in seeing the positives in her and the other students. Many said unencouraging things to her, such as “you are always late,” “your behavior is unacceptable,” and “your parents must come to school.” On the other hand, I always told her positive things - that she is smart, can try, and has reading and writing abilities. I instilled motivation in her.”

Rania's systematic approach involved consistent affirmation patterns, explicit counter-narratives to the deficit messages Reem received from others, a focus on capabilities rather than failures, and high expectations combined with genuine support. This was not personality-based warmth; it was methodical professional practice. The principal's repeated inquiries about “what exactly” Rania did suggested that her success seemed mysterious to those who viewed relationship-building as a natural talent rather than a learnable skill.

The depth of the relationship that emerged enabled comprehensive student support. Rania became a trusted adult figure for Reem, who confided in personal struggles related to family, peers, and school. This trust did not develop spontaneously; it resulted from Rania's systematic demonstration that she was safe, consistent, and genuinely invested in Reem's success.

Across these three narratives, a consistent pattern emerges: contextual intelligence and relational pedagogy operate as integrated capabilities rather than separate skills. Pre-service teachers who developed sophisticated environmental reading paired it with systematic relationship-building approaches. They recognized that complex contexts require complex responses—not just understanding what is happening, but creating methodical interventions that address root causes while building authentic connections. This integration of environmental sensitivity with systematic relational practice represents a form of professional expertise that current teacher education approaches rarely name explicitly, much less deliberately cultivate.

### 4.3 From compliance to innovation

This theme reveals how pre-service teachers transition from rule-following, survival approaches to principled, innovative practice during their internships. This transformation consistently involved a strategic departure from conventional methods that prioritized institutional compliance over student success, representing a fundamental shift in professional identity and practice philosophy.

#### 4.3.1 Rejecting ineffective conventional approaches

Participants consistently discovered that standard approaches failed their most challenging students, leading them to develop creative alternatives. Lina's experience with a child from a problematic home environment illustrates this pattern:

“In my class, there is a 7-year-old boy who comes from a difficult home environment. His parents have mental health issues, and he often displays aggressive behavior and uses foul language. However, I have found that playing with sand helps to calm him down. So, I made him a small sandbox and appointed him as the ‘policeman' of the yard. This helped him to learn more respectful language and improve his behavior.”

Lina's innovation violated conventional wisdom about classroom management. Standard behavioral approaches would focus on consequences for aggression and foul language—punishment, time-outs, and behavior charts. Instead, Lina implemented environmental modifications to address the child's underlying regulatory needs. The sandbox provided sensory regulation, while the “policeman” role built self-esteem by focusing on responsibility rather than on deficits. This represents principled innovation rather than arbitrary rule-breaking: Lina assessed the child's actual needs and designed responses accordingly.

Her approach became increasingly sophisticated as she identified additional opportunities for strength-based intervention:

“I also noticed that he loves to ride his bicycle, so I used that to encourage good behavior. Every time we go outside, I let him ride his bike first and praise him in front of the other children for his riding skills. I even taught him some traffic signs and placed them on the path so he could ride safely and in accordance with the rules. Moreover, when he gets angry, I create a ‘curse box' for him to use. This has helped him to control his emotions and avoid using foul language. Since I started using these interventions, the boy has become much calmer and better behaved in class.”

Note the systematic integration of multiple unconventional elements: public recognition of strengths rather than a focus on problems, environmental design that supports regulation rather than punishment, creative outlets for strong emotions (the “curse box”), and teaching responsibility through interest-based activities. Each innovation departed from standard practice, yet each was carefully designed to serve authentic student needs. The phrase “since I started using these interventions” suggests that Lina recognized her approach as a deliberate methodology rather than as spontaneous kindness.

#### 4.3.2 Investigating root causes over surface solutions

Sometimes the transition from compliance to innovation involves recognizing when conventional approaches are not only ineffective but also actively contribute to failure. Samia's experience illustrates this deeper level of principled innovation:

“I remember meeting Othman, a quiet 5-year-old kid. He had difficulty differentiating and naming colors, and his abilities were clearly below those of his peers. I tried my best to teach him, but we had to start from scratch every morning, and Othman's lack of confidence made it even more challenging. One day, after researching his case and speaking with his mother, I realized she was associating colors with English words instead of teaching them in Arabic. I knew this confused him, so I partnered with his mother.”

Samia's innovation began with investigation rather than intensification. When standard color-teaching methods repeatedly failed, she did not simply try harder; she questioned whether the problem lay in the method itself. Her discovery that homeschools' language inconsistency was creating confusion led to systematic innovation:

“Together, we shared class activities and tools, using tangible objects like oranges for the color orange and cucumbers for green. We also sang color songs and used apps to support Othman's learning. Over time, he improved his abilities and gained greater confidence. It was amazing to see him finally learn to name, remember, and differentiate all the colors without additional aids. This experience taught me the power of collaboration between parents and teachers and the importance of finding innovative ways to teach children.”

Samia's approach violated several conventional practices: partnering with parents as co-teachers rather than maintaining teacher authority; using multiple languages for instruction rather than enforcing a single-language policy; employing real objects rather than relying on abstract teaching materials; and coordinating home-school practices rather than expecting home to conform to school. The student justified each departure from standard practice based on the need rather than institutional convenience. Her explicit recognition that this “taught me the power of collaboration” suggests a growing awareness that innovation entails systematic relationship-building rather than merely creative teaching techniques.

#### 4.3.3 Challenging exclusionary institutional practices

The most profound innovations sometimes involve challenging not just ineffective methods but exclusionary assumptions about which students can participate in which activities. Suzan's experience demonstrates this dimension of principled innovation:

“I had a challenge when I took over the special education class and met Haya as a new teacher... Haya, who had autism, imposed her conduct on the class. The former teacher and assistant tolerated her condition, but I had to get Haya to interact with the other pupils... It took a lot of patience, persistence, and discipline to get Haya out from under tables and follow classroom regulations. I stopped her from escaping during sleep. Haya began interacting with her peers at breakfast/lunch, and the principal noted a decrease in her crying.”

Suzan's initial interventions challenged the previous approach of tolerance through separation. By insisting on Haya's participation in regular classroom routines, she rejected the assumption that accommodation meant accepting isolation. This required patience, persistence, and discipline, not directed at the child as punishment, but as systematic support to help Haya develop capabilities that previous teachers had not anticipated.

Her most significant innovation involved challenging exclusionary practices around special events:

“The hardest part was encouraging Haya to attend the Mother's Day party and prepare a gift for her mom, but I was determined to help. Haya attended the party and gave her mom a gift with encouragement and assistance. This was Haya's big win, and we were proud. This taught me that everyone has a unique path, and it is crucial to find the perfect one to help them achieve.”

Suzan's innovation was not just including Haya in the party; it was refusing to accept that some students could not participate in meaningful community events. Her determination “to help” Haya attend represents principled innovation at its clearest: departing from low expectations not recklessly, but through systematic support that combines encouragement and assistance. The phrase “everyone has a unique path” captures her emergent philosophy: innovation means finding what works for each student rather than expecting students to conform to predetermined approaches.

Across these three narratives, the transition from compliance to innovation follows a consistent pattern. Pre-service teachers recognized when standard approaches were not working, investigated root causes rather than intensifying failed methods, designed alternatives grounded in student assessment rather than institutional convenience, and persisted despite resistance or skepticism from others. Critically, their innovations were not random experimentation; they represented thoughtful, ethical departures from convention when convention failed students.

This pattern challenges assumptions underlying much teacher preparation, which often emphasizes compliance with established practices as evidence of professional competence. These success stories suggest something different: that genuine professional competence sometimes requires principled innovation that prioritizes student success over institutional conformity. The college internship experience, with its extended duration and supportive supervision, provided a space in which beginning teachers could develop both the courage to depart from convention and the judgment to do so responsibly.

## 5 Discussion

These findings make three theoretical contributions to understanding how innovative teaching capabilities develop during teacher preparation. First, they reveal the systematic mechanisms by which Educational Eros operates in practice, showing how personal vulnerability is deliberately deployed through daily pedagogical routines rather than remaining an abstract, passionate commitment. Second, they challenge assumptions about novice teacher capabilities within ecological frameworks by documenting sophisticated multi-system navigation that traditional applications of Bronfenbrenner's theory rarely attribute to beginning practitioners. Third, they reconceptualize autonomy within Self-Determination Theory, demonstrating how autonomous decision-making in teaching manifests as principled innovation and thoughtful rule-breaking in service of student needs, rather than simply choosing among predetermined options.

The Israeli context offers theoretical insights into the development of innovation during teacher preparation. Pre-service teachers navigate culturally diverse settings where Arab and Jewish students prepare for teaching in either mixed or segregated schools. This diversity creates immediate demands for cultural competence rather than treating it as an abstract ideal. Flora's transformation from cultural bias to effective cross-cultural teaching illustrates this pattern. Her initial discomfort working with an Arab student, followed by a breakthrough connection, reflects how cultural diversity in Israeli teacher preparation may accelerate the development of contextual intelligence. When pre-service teachers cannot avoid cross-cultural encounters, personal biases become pedagogical obstacles requiring immediate transformation. The year-long internship structure provides extended time for such transformations to occur through sustained relationship-building rather than brief multicultural exposure.

### 5.1 Extending educational Eros theory

The transformation of personal adversity into professional capability evident across participants' narratives challenges prevailing conceptualizations of teacher preparation as primarily technical skill acquisition (Flores, 2020; Le Huu Nghia and Ngoc Tai, 2017; Cai et al., 2022; Meyer et al., 2023; Neander Christensson, 2024), extending conventional applications of Educational Eros theory in important ways.

Tsabar's (2014) notion of Educational Eros operates through systematic mechanisms that involve the deliberate deployment of personal vulnerability in the service of student growth, supporting Craig's (2022) emphasis on passionate commitment while extending Main's (2012) work on educational encounters.

What participants such as Hila and Jameel demonstrated goes beyond the conceptual descriptions typically found in Educational Eros literature; they showed us the mechanisms by which passionate commitment translates into innovative practice.

Hila's daily letter-writing ritual with her bereaved student was not spontaneous emotional support; it was methodical professional practice grounded in personal understanding. Jameel's explicit articulation that “I see myself in those pupils” captures what Educational Eros looks like when it becomes conscious professional methodology rather than remaining implicit caring. This extends Tsabar's (2014) theoretical work by providing empirical documentation of how Educational Eros operates: through strategic vulnerability (sharing survival, not just pain), through systematic ritual (daily practices, not occasional gestures), and through deliberate community building (extending care beyond individual relationships).

The theoretical implication is significant: Educational Eros should not be conceptualized primarily as a personality trait or emotional disposition that some teachers naturally possess. Instead, these findings suggest it can be understood as a learnable professional practice involving specific, identifiable techniques that intensive field experiences can systematically cultivate.

### 5.2 Challenging ecological theory assumptions

Participants' success narratives revealed environmental responsiveness that challenges assumptions about novice teacher capabilities within ecological frameworks. Their ability to read complex social, emotional, and cultural environments, exemplified by Hiba's recognition of intersecting family illness, sensory processing challenges, and peer dynamics, suggests capabilities surpassing what traditional applications of Bronfenbrenner's (1979) ecological theory typically attribute to beginning practitioners (Elliott and Davis, 2020; Tong and An, 2024).

What makes this theoretically interesting is how these pre-service teachers navigated multiple system levels simultaneously rather than sequentially. Ecological Systems Theory typically suggests that understanding environmental influences requires developmental maturity and extensive experience. However, participants demonstrated sophisticated ecological intelligence during their first sustained teaching experiences, reading how microsystem factors (peer relationships), mesosystem connections (home-school dynamics), and macrosystem influences (cultural expectations) intersected to create student difficulties.

Hiba did not just notice that Sami had sensory processing challenges, or that his mother was ill, or that peers excluded him. She recognized how the three layers interacted and designed interventions that addressed multiple levels of the system simultaneously through transparent community dialogue. This suggests that ecological intelligence may develop more rapidly through intensive field experiences than current theoretical applications assume, particularly when supportive supervision explicitly encourages multi-system thinking.

This evidence extends recent research calling for more sophisticated ecological theory applications by demonstrating how pre-service teachers simultaneously navigate multiple system levels, family, school, and cultural contexts, rather than addressing isolated components, while supporting contemporary work emphasizing systematic approaches to emotional engagement in educational practice (Artola Bonanno et al., 2024; Borremans et al., 2024; Wang, 2023; De Lisio et al., 2025).

### 5.3 Reconceptualizing autonomy in self-determination theory

The theme of principled innovation extends the application of Self-Determination Theory in teacher education by revealing how autonomy manifests differently from its typically conceptualized form. The systematic innovations described by participants, Lina's creation of sensory regulation tools, Samia's multilingual pedagogical approach, Suzan's challenge to exclusionary practices, demonstrated autonomous decision-making that created entirely new professional responses while maintaining ethical accountability to student wellbeing (Deci and Ryan, 2000; Ryan and Deci, 2020; Brenner, 2022; Chiu et al., 2024; Yang et al., 2025).

Self-Determination Theory typically conceptualizes autonomy in educational contexts as choice among alternative students choosing which assignment to complete, and teachers choosing which instructional strategies to employ. However, the autonomy evident in these success stories looks quite different. Participants were not choosing among predetermined options provided by teacher education programs or cooperating teachers. They were creating entirely new responses when conventional options failed their students.

Lina's sandbox solution, Samia's parent partnership approach, and Suzan's inclusion innovations were not selections from a menu of approved strategies. They were principled departures from convention, justified by careful assessment of student needs and grounded in ethical commitment to student success. This suggests that we need expanded conceptualizations of autonomy in teacher education that include the capacity for thoughtful rule-breaking in the context of failing students.

The theoretical implication challenges how teacher preparation programs typically operationalize autonomy support. If we focus solely on providing pre-service teachers with choices among established practices, we may constrain the innovative autonomy these success stories reveal. Proper autonomy support may require explicitly encouraging appropriate departures from convention when student needs demand it, which, in turn, requires supervisors who can distinguish between principled innovation and reckless experimentation.

### 5.4 Toward a conceptual framework

Based on these theoretical contributions, we propose a framework for understanding how innovative capability develops during intensive field experiences. This framework integrates insights from all three theoretical perspectives rather than treating them as separate explanatory systems.

Innovation emerges through dynamic interaction among four elements. First, personal resources, life experiences, cultural knowledge, and hard-won wisdom from navigating difficulty provide raw material for innovation. However, these resources do not automatically become professional capabilities. Their transformation requires supportive environmental conditions (the ecological dimension): extended time for relationship-building, supervision that encourages personal-professional integration, and institutional tolerance for thoughtful experimentation.

Within such environments, psychological empowerment becomes possible (the SDT dimension). Pre-service teachers need autonomy to depart from conventional approaches when they are not working, competence that builds through successful student breakthroughs, and deep relatedness that develops through intensive one-on-one work. However, environmental support and psychological empowerment still do not guarantee innovation. The fourth element, passionate commitment (Educational Eros), provides the motivational force. This deep care makes someone willing to be professionally vulnerable, to take appropriate risks, to persist when easier paths exist. The framework suggests that innovation requires simultaneous attention to all four dimensions: personal resources, supportive environments, psychological empowerment, and passionate commitment, which together yield innovative teaching approaches. Remove any element, and innovation becomes unlikely. Participants who successfully transformed personal experience into professional capability did so within supportive internship environments that satisfied their psychological needs, while their passionate commitment to students drove persistent innovation.

This integrated framework helps explain why partial interventions in teacher preparation often produce limited results. Programs emphasizing reflection without supportive field environments will not generate innovation. Supportive environments that neglect psychological needs will not suffice. Even under ideal conditions, innovation will not occur in the absence of passionate commitment. The theoretical contribution is to recognize that innovation in teacher preparation requires simultaneous attention to personal, environmental, psychological, and motivational dimensions, rather than focusing on any single factor.

Our findings both align with and extend several important lines of contemporary research. The results directly address an important gap identified by Liu et al.'s (2024) comprehensive review, that teacher innovation research has focused primarily on experienced teachers rather than those in preparation. Our documentation of the emergence of innovation in participants' internship success stories provides empirical evidence for recent calls to recognize pre-service teachers' innovative potential (Fitzsimons et al., 2024; Liu et al., 2024; Han and Abdrahim, 2023). The transformation processes revealed support for arguments challenging deficit perspectives and provided specific mechanisms through which pre-service teachers develop innovative capabilities during authentic field experiences.

The systematic relational pedagogy evident in participants' success stories extends contemporary research on factors influencing creativity and narrative approaches of teacher education students. Recent work demonstrates clear benefits of classroom-based storytelling and personal narrative integration (Spencer and Pierce, 2023; Browning and Hohenstein, 2024; Shi and Cheung, 2024; Landrum et al., 2019), but our analysis reveals how personal narratives become systematically integrated into professional methodology during intensive field experiences rather than simply enhancing existing practices. This represents a deeper level of integration than current literature typically documents. Contemporary research confirms that teacher-student relationships significantly impact academic engagement through social support mechanisms (Liu, 2024).

However, the innovative capabilities revealed in these narratives challenge assumptions in contemporary literature, emphasizing primarily the overwhelming challenges faced by beginning teachers. While extensive research documents significant difficulties during the novice years, including classroom management struggles, inadequate support systems, and high attrition rates (Kozikoglu, 2019), our analysis of participants' most meaningful successes suggests that deficit-focused approaches may systematically overlook the innovative capabilities that pre-service teachers bring to practice.

The sophisticated contextual intelligence and principled innovation documented in these success narratives challenge linear development models from novice to expert, supporting recent research on pre-service teacher agency and transformative capacity (Chen-Levi et al., 2024; Fitzsimons et al., 2024; Zhang et al., 2024).

## 6 Implications

These findings fundamentally challenge how we approach teacher preparation. By focusing on success rather than struggle, we revealed innovative capabilities that deficit-focused research systematically overlooks. The success stories suggest we have been asking the wrong questions about pre-service teachers, constantly focusing on “what skills are they lacking?” when we should be asking “what innovative potential already exists and how do we cultivate it?” This reconceptualization carries implications at multiple interconnected levels: curriculum design, field experience structures, supervision practices, and institutional policies.

## 7 Curriculum and pedagogy

The transformation of personal experience into professional capability, as evidenced by participants' success stories, suggests that teacher education curricula require explicit attention to integrating personal and professional dimensions. This is not simply about encouraging reflection, but rather about creating structured opportunities for pre-service teachers to examine their life experiences and consider systematically how these might become professional resources through supportive supervision that encourages appropriate vulnerability while maintaining professional boundaries (Arnsby et al., 2023; Sydnor et al., 2023; Butler et al., 2023). When Hila transformed her childhood grief into a comprehensive approach to supporting bereaved students, or when Lili converted her experiences of classroom exclusion into sophisticated strategies for including anxious children, they were not simply drawing on natural empathy. They were engaging in complex integration work that teacher education programs could support more deliberately.

This suggests several concrete curricular shifts. Teacher preparation programs might develop explicit coursework or workshop sequences focused on what we might call “contextual intelligence, the capacity to read complex social, emotional, and cultural environments and respond with contextually appropriate interventions. This approach extends beyond traditional classroom management training to encompass systematic attention to pattern recognition across multiple cases, environmental assessment strategies that consider intersecting systems, and intervention design that addresses root causes rather than surface symptoms (Tong and An, 2024; Almughyiri, 2025; Renn and Smith, 2023). The success stories revealed that pre-service teachers who developed this capacity were those who received sustained support in noticing subtle cues, interpreting their meaning within broader contexts, and designing responses that acknowledged complexity.

Similarly, relationship-building and emotional connection should be reframed as systematic professional skills rather than as personality traits that some people naturally possess. The relational pedagogy evident in participants' success stories, Rania's methodical affirmation practices, Salam's integration of cultural resources, and Hiba's deliberate community-building demonstrates that care and connection can be structured professional approaches that are teachable, practiceable, and refinable. This challenges traditional distinctions between technical and relational competencies while supporting contemporary research on the development of teacher-student relationship competence (Borremans et al., 2024; Artola Bonanno et al., 2024; Liu, 2024; Li and Zhang, 2024).

## 8 Field experience structures

The field experience structure itself requires careful reconsideration in light of these findings. The year-long internship that characterized the Israeli context provided something that shorter field experiences may not: adequate time for authentic relationship-building, for making mistakes and recovering from them, for trying innovative approaches and refining them based on student response. When we examine the success stories closely, we see that transformation did not happen quickly. Flora's journey from cultural bias to effective cross-cultural teaching unfolded over months of sustained engagement. Suzan's work with Haya involved persistent, incremental progress rather than an immediate breakthrough.

This temporal dimension has important implications for how we structure field experiences internationally. While not every context can implement year-long internships, the principle remains: pre-service teachers need sufficient time to move beyond survival mode into genuine innovation. Field experiences should be designed to provide multiple opportunities for risk-taking, developing relationships that are deep enough to support authentic vulnerability, and experimenting with approaches that might initially feel uncomfortable or uncertain. This aligns with recent research emphasizing the importance of sustained, well-structured field experiences in developing teacher effectiveness (Fairbrother et al., 2025; Sohdi, 2025; Stevens et al., 2024).

The cultural diversity inherent in the Israeli teacher education context offers another important insight. When pre-service teachers cannot avoid cross-cultural encounters, and diversity is an immediate pedagogical reality rather than an abstract ideal, personal biases and assumptions become obstacles that require immediate transformation. This suggests that field placements should deliberately seek authentic multicultural contexts in which cultural competence is necessary rather than optional, and in which pre-service teachers must develop contextual intelligence to be effective.

## 9 Supervision and mentoring

Perhaps the most critical implication concerns supervision and mentoring during field experiences. The success stories revealed that innovation emerged not despite supervision, but through it, when supervisors created environments that supported appropriate professional vulnerability while maintaining clear boundaries and ethical standards. This requires a fundamental shift in how we prepare and support those who supervise pre-service teachers.

Supervisors need development in facilitating personal-professional integration conversations, dialogues that help pre-service teachers identify potentially relevant life experiences, examine them critically for professional applicability, and develop systematic ways to deploy personal wisdom in the service of student needs. This is delicate work requiring supervisors who can distinguish between appropriate sharing that enhances professional capability and inappropriate boundary-crossing that compromises professional relationships.

Equally important, supervisors need frameworks for distinguishing between principled innovation and reckless experimentation. The success stories demonstrated that practical innovation was never random rule-breaking but rather a thoughtful departure from conventional approaches when those approaches failed to serve student needs. Lina's creation of sensory regulation tools, Samia's development of multilingual pedagogical strategies, and Suzan's challenge to exclusionary practices all represented carefully considered innovations grounded in student assessment and responsive to authentic needs. Supervisors need support in recognizing such thoughtful innovation, encouraging its development, and helping pre-service teachers reflect on the ethical dimensions of their choices (Ewing, 2021; Qin, 2025; Murray et al., 2025).

This transformation in supervision requires substantial faculty development initiatives. Teacher educators need opportunities to examine their own assumptions about the relationship between compliance and quality, to develop strength-based approaches that identify innovative capabilities rather than focusing primarily on deficit remediation, and to practice the kind of supportive vulnerability they are asking pre-service teachers to embrace.

## 10 Institutional policies

At the institutional level, the success stories suggest that innovation flourishes when organizational cultures genuinely value creative problem-solving in service of student success, rather than merely complying with established practices. This requires policy frameworks that explicitly support appropriate risk-taking during internships while providing clear ethical guidelines that distinguish between beneficial innovation and harmful deviations from evidence-based practice.

The strong college-school partnerships evident in the Israeli context proved essential for creating environments where pre-service teachers felt supported to experiment. When cooperating teachers, college supervisors, and school administrators shared a commitment to nurturing innovative practice, pre-service teachers received consistent messages about the value of creative problem-solving. This suggests that teacher education institutions need substantial investment in partnership development, not just placement agreements, but genuine collaborative relationships focused on shared goals for pre-service teacher development.

## 11 International adaptation

While these findings emerged from a specific cultural and institutional context, the underlying developmental processes, personal-professional integration, contextual intelligence development, and principled innovation appear to represent fundamental aspects of professional growth that transcend particular national or cultural settings. What varies across contexts are the structural enablers: the time allocated for relationship-building, the quality and intensity of supervision, the institutional tolerance for innovation, and the cultural expectations surrounding teacher authority and student-teacher relationships.

This suggests that international adaptation requires thoughtful consideration of how these structural elements can be effectively implemented within different educational systems. Countries with shorter field-experience requirements might compensate by providing more intensive supervision or by earlier integration of personal-professional reflection into coursework. Systems with more hierarchical educational cultures may require explicit efforts to create safe spaces for innovative thinking. The key insight is that the innovative capabilities demonstrated in these success stories are not culturally specific gifts but developmental achievements that supportive environments can nurture across diverse contexts.

However, the Israeli context, with its emphasis on year-long internships and strong college-school partnerships, may present challenges for direct transferability to other educational systems with different structural arrangements, resource allocations, and cultural expectations (Zhang et al., 2024; Dvir and Schatz-Oppenheimer, 2020; Guberman et al., 2024). Educational systems could carefully consider how to adapt these insights within their specific constraints and possibilities.

## 12 Limitations and future research

Several limitations suggest important directions for future investigation that could enhance understanding of these developmental processes. Our focus on particularly successful experiences shared by pre-service teachers, while providing rich examples of innovative practice development, may not represent the full range of internship experiences and potentially overlooks factors that prevent innovative capability development in more typical situations. The Israeli context, with its emphasis on year-long internships and strong college-school partnerships, may limit the transferability of its approaches to other educational systems with different structural arrangements, resource allocations, and cultural expectations (Zhang et al., 2024; Dvir and Schatz-Oppenheimer, 2020; Guberman et al., 2024).

Future research should examine how these themes manifest across different levels of success and various types of challenges, supporting recent calls for more comprehensive investigations of pre-service teacher development across diverse contexts and populations (Kaur, 2024; Zaman and Mahanta, 2025). Longitudinal studies investigating how innovative capabilities evident in internship success stories translate into continued professional growth and career-long innovation would provide crucial insights into the sustainability of these developmental processes and their long-term impact on teaching effectiveness and student outcomes.

Comparative studies examining how different institutional contexts, cultural settings, student populations, and subject areas influence the development of contextual intelligence, relational pedagogy, and principled innovation would enhance understanding of these patterns' generalizability and cultural specificity. Additionally, research exploring multiple stakeholder perspectives, including cooperating teachers, college supervisors, and students, on the innovative approaches described in pre-service teachers' success narratives would provide valuable insights into the factors that support or constrain the development of creative practice during field experiences.

## 13 Conclusions

Analysis of pre-service teachers' most meaningful success stories reveals them as potentially sophisticated professional practitioners whose innovative capabilities may be systematically underdeveloped by current teacher education approaches that prioritize deficit remediation over the cultivation of strengths. The three themes that emerged from these success narratives suggest that adequate teacher preparation requires a fundamental reconceptualization of the relationships among personal experience and professional competence, technical skills and adaptive expertise, and compliance and innovation in professional practice.

These success stories demonstrate that conditions enabling the innovative practices evident in transformative teaching experiences, including extended time for authentic relationship-building, supportive supervision that encourages personal and professional integration, and institutional tolerance for appropriate risk-taking, represent resource-intensive approaches that challenge conventional models of teacher preparation. Nevertheless, the transformative potential revealed through participants' accounts of passionate, committed encounters with authentic opportunities to impact students' lives suggests that such investments may be essential for preparing teachers capable of addressing contemporary educational challenges and diverse student needs.

The insights drawn from these success narratives suggest that teacher education approaches should recognize and systematically cultivate the creative power evident in intensive field experiences, while acknowledging the sophisticated supervisory capabilities and institutional support structures necessary to facilitate such development. The challenge lies in creating systematic approaches to cultivating the innovative potential demonstrated in these success stories that maintain the authenticity, vulnerability, and relationship-centered focus that appear central to transformative practice development, while ensuring that such approaches can be implemented ethically and sustainably across diverse institutional contexts.

## Data Availability

The raw data supporting the conclusions of this article will be made available by the authors, without undue reservation.
